# Heavy Metal Contamination of Soils around a Hospital Waste Incinerator Bottom Ash Dumps Site

**DOI:** 10.1155/2016/8926453

**Published:** 2016-02-29

**Authors:** M. Adama, R. Esena, B. Fosu-Mensah, D. Yirenya-Tawiah

**Affiliations:** ^1^Institute for Environment and Sanitation Studies, University of Ghana, Legon, Ghana; ^2^School of Public Health, University of Ghana, Legon, Ghana

## Abstract

Waste incineration is the main waste management strategy used in treating hospital waste in many developing countries. However, the release of dioxins, POPs, and heavy metals in fly and bottom ash poses environmental and public health concerns. To determine heavy metal (Hg, Pb, Cd, Cr, and Ag) in levels in incinerator bottom ash and soils 100 m around the incinerator bottom ash dump site, ash samples and surrounding soil samples were collected at 20 m, 40 m, 60 m, 80 m, 100 m, and 1,200 m from incinerator. These were analyzed using the absorption spectrophotometer method. The geoaccumulation (*I*_geo_) and pollution load indices (PLI) were used to assess the level of heavy metal contamination of surrounding soils. The study revealed high concentrations in mg/kg for, Zn (16417.69), Pb (143.80), Cr (99.30), and Cd (7.54) in bottom ash and these were above allowable limits for disposal in landfill. The study also found soils within 60 m radius of the incinerator to be polluted with the metals. It is recommended that health care waste managers be educated on the implication of improper management of incinerator bottom ash and regulators monitor hospital waste incinerator sites.

## 1. Introduction

Waste incineration is the main strategy used in treating hospital waste in many developing countries including Ghana. While this has the advantage of killing pathogens in the waste stream and reducing waste volume and reactivity, incineration has been found to impact on the environment through the release of pollutants in the emissions of ash which has environmental and public health implication [1]. Industrialized countries such as Netherlands, United States, and Germany have phased out the use of incineration for hospital waste management and have moved to treat hospital waste through the use of autoclave, microwave, and recycling as a way of mitigating health and environmental consequences [2].

Even more challenging for hospital waste management in developing countries is the expanding health service industry with expanding opportunities for waste incineration which provides an interim solution to managing hospital waste [3]. A major concern of hospital waste incineration is the generation of persistent organic pollutants (POPs), such as polychlorinated biphenyls (PCBs), dioxins, polycyclic aromatic hydrocarbons (PAHs), and other cancer causing organics [3, 4]. Hospital waste incineration is known to not completely destroy the metallic components of the waste stream but rather concentrate heavy metals into the bottom ash [5]. Thus incinerated hospital waste bottom ash has more heavy metals (chromium, cadmium, lead, mercury, zinc, and other metals) as well as organic compounds (PCBs, dioxins, benzene, and other cancer causing organics) which if not well disposed of can pollute the environment and pose public health problems such as acute respiratory syndromes, gastrointestinal abnormalities, and various cancers [6–8]. Because of these environmental concerns, incinerator bottom ash management has been under continuous scrutiny and control [9] in recent times.

According to Ghana's healthcare waste management guideline, waste incineration is approved for use for infectious and hazardous waste. It stipulates that incinerator bottom ash should be disposed of at landfilled sites and should not be deposited or scattered on the surface of open dumps and must be protected against access by scavengers [10]. However, uncontrolled disposal of ash is a common practice in the country. To demonstrate the effect of heavy metal pollution in hospital soils and bring to attention the need for sustainable management of the incinerator ash, this study was conducted in one of Ghana's Teaching Hospitals to assess the soil pollution levels of surrounding surface soils from the incinerator.

## 2. Materials and Methods

**Figure 1:**
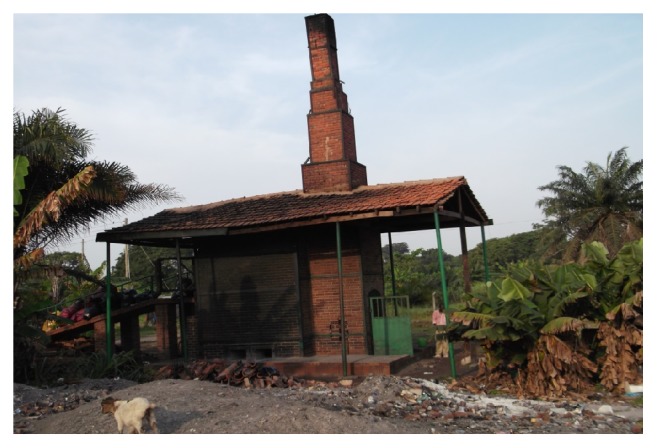
Showing the incinerator and the ash dumping point.

The study was conducted in one of Ghana's Teaching Hospitals with a bed capacity of almost 2000. The hospital generates on the average 2.8 kg/bed/day of clinical waste resulting in about 17.5 tons of clinical waste produced in a week. The hospital uses a locally built small-scale De Montfort type that has an in-built drier that could dry wet waste very fast and a burning chamber for five tons of waste which could burn completely within three hours. Adjacent to the incinerator is the ash dump site where the bottom ash after incineration is disposed of (Figure 1).

### 2.1. Study Design

A cross sectional study design was employed in this research. Bottom ash from the incinerator and soil samples within a 100 m radius at given interval of 20 m and 1,200 m away from the incinerator were collected and analyzed for cadmium (Cd), chromium (Cr), silver (Ag), mercury (Hg), lead (Pb) and zinc (Zn). Data collection was done from October 2012 to January 2013.

### 2.2. Sample Collection

Bottom ash samples were collected twice weekly from the hospital waste incinerator using the quartering method. Soils were sampled at six different sampling points away from the incinerator. Five of the sampling points were within 100 m from the incinerator (interval between sampling points were 20 m apart) whereas the sixth point was 1,200 m from the incinerator. At each distance from the incinerator, three samples of soil were randomly taken with each sample made up of 5 composites (well mixed) using soil auger into labelled plastic bags. About 30 g of the sample was collected into a zip lock bag for laboratory analysis.

The auger was washed with water and rinsed with distilled water and dried after sampling at each point to avoid cross contamination. All samples were analyzed at the Council for Scientific and Industrial Research (CSIR) Metals laboratory.

### 2.3. Bottom Ash and Soil Sample Preparation

In the laboratory, bottom ash was collected and sieved with 2 mm mesh sieves to separate the various particle sizes from the fine ash. A 5 g of bottom ash sample was weighed into a 100 mL polytetrafluoroethylene (PTFE) Teflon beaker which was previously acid washed. Two milliliters of 65% nitric acid (HNO_3_) and 5 mL of 36% hydrochloric acid (HCl) were added to each sample in a fume chamber. The samples were then loaded on a microwave carousel. The vessel caps were secured tightly using a wrench. The complete assembly was microwave irradiated for 20 minutes using milestone microwave lab station ETHOS 900, INSTR: MLS – 1200 MEGA. 6.

Similarly, 5 g of soil samples was digested with 60% perchloric acid (HClO_4_) (BDH Chemicals Ltd., UK), concentrated nitric acid (HNO_3_) (BDH Chemicals Ltd., UK), and sulphuric acid (H_2_SO_4_) (BDH Chemicals Ltd., UK). Blanks were prepared to check for background contamination by the reagents used.

### 2.4. Heavy Metal Analysis

The digested samples were analyzed for the heavy metals Cd, Cr, Ag, Pb, and Zn using atomic absorption spectrophotometer Agilent 240FS in the flame mode and cold vapour mode for Hg.

### 2.5. Geoaccumulation Index (*I*_geo_) Assessment

The index of geoaccumulation (*I*_geo_) is used to evaluate the contamination by comparing the levels of metal obtain to the background levels originally used with bottom sediment [11, 12]. The index is then calculated using the equation:

(1) $$\begin{array}{c} I_{geo}={log}_{2}\left(\;\frac{C_{n}}{1.5B_{n}}\;\right), \end{array}$$

where *C*_*n*_ represents the measured concentration of heavy metal in the soil sample. *B*_*n*_ is the geochemical background concentration of the heavy metal [12] (Table 1).

### 2.6. Pollution Load Index (PLI)

Estimation of pollution load index was done using Tomlinson's pollution load index (PLI) [13]. This was calculated using the heavy metal data and metal concentration for the world shale (abundance of element in earth's upper continental crust) average as the background value [14]. The PLI of soils was calculated by obtaining the *n*th root from the *n* number of obtained contamination factor (CF) for all the metal [15]:

(2) $$\begin{array}{c} PLI=\sqrt[{n}]{{CF}_{1}\times {CF}_{2}\times {CF}_{3}\times \cdots \times {CF}_{n}}, \end{array}$$

where *n* is the number of heavy metals and CF = *C*_metal_/*C*_background_. Categorization of PLI used is presented in Table 2.

### 2.7. Quality Control

Blanks were used to check contamination during sample preparation and analysis. Reference standards were used for the elements of interest; blanks and samples were digested under the same conditions. Reference standards used are from FLUKEA ANALYTICAL, Sigma-Aldrich Chemie GmbH, products of Switzerland. Before the analysis, equipment was calibrated using the appropriate standards.

### 2.8. Data Analysis

The data was analyzed using SPSS software version 16.0. Descriptive statistics were run to determine mean and standard deviations of detected metals. Detected levels of heavy metals in bottom ash were compared with USEPA acceptable limits whereas levels of heavy metals in soils compared with the critical allowable limits of heavy metals in soils [16, 17]. Pearson correlation analysis was used to assess the correlation between the metals in the soil.

## 3. Results and Discussion

All heavy metals assessed in bottom ash were within detectable limits.  Table 3 shows the mean of heavy metals detected in bottom ash. In a descending order of concentration Zn > Pb > Cr > Ag > Cd > Hg were all found to be above USEPA allowable limits for safe disposal to a landfill site. Our findings were similar to studies conducted in Morocco and Kenya [18, 19]. The heavy metals found in the bottom ash are usually associated with the waste feed stock (thermometers, blood pressure cuffs, laboratory chemicals, plastics, syringes, etc.) or construction material of the incinerator [20]. The high levels of Zn may be due to the clay used to make the bricks from which incinerators were built and the fact that many of the medical items are made of metal alloys such as Zn and Ti [6, 20].

Ghana's Medical Waste Management Guidelines advocate for controlled disposal of hospital incinerator bottom ash at engineered landfills. This policy indirectly supports the need for periodic monitoring of hospital incinerator bottom ash quality, environmental pollution potential, and treatment strategies to ensure the safety of bottom ash to be disposed of [10]. Observations made at the waste incinerator site showed continuous dumping of bottom ash in an open dump pit close to the incinerator and this may be attributed to the heavy metal levels observed in soils within 100 m from the incinerator (Table 4). This study found the levels of Pb, Zn, Hg, and Cd to be above acceptable limits in the soil. Heavy metal levels determined in soils at 1,200 m from the incinerator were all below the acceptable limit except for mercury that was slightly higher (0.5 mg/kg) than the acceptable limit (0.3 mg/kg). As expected, there was generally a decrease in heavy metal concentration with increasing distance from incinerator. Hg and Zn were above critical levels between 20 and 80 m while Pb and Cd levels were above critical levels between 20 and 40 m.

A recent paper reported that metals, such as Pb, Cr, Cd, Cu, and Zn in bottom ash from a medical waste incinerator were with high leachability [21]. Leaching of metals into soils can be associated with their cation exchange capacity, complexing organic and retention capacity of oxides and carbonates in the soil. It is also observed that, so far as source of heavy metal contamination of soil remained active and continuous, soil contamination levels will increase [22].

All the heavy metals (Hg, Pb, Zn, Ag, Cr, and Cd) recorded strong positive correlation to each other (*p* < 0.05) except for Cr and Hg where a negative correlation (−0.573; *p* = 0.05) was observed (Table 5). The strong correlations observed indicate that each of the paired elements in the soil has common contamination sources which in this case may be linked to the open dumping of incinerator ash. The negative correlation between Hg and Cr may be as a result of the changes in their chemical forms (speciation) and bioavailability [23].

**Figure 2:**
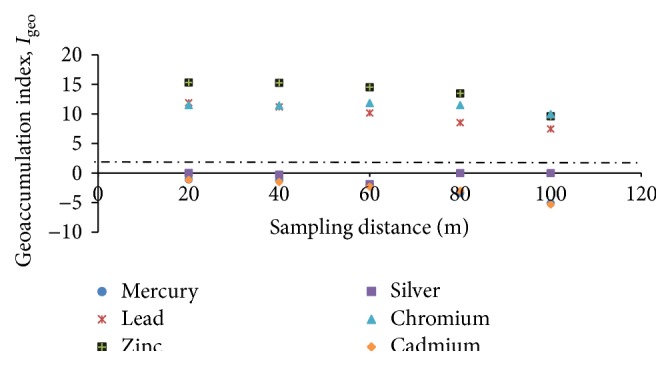
Soil contamination levels at sampled distances.

**Figure 3:**
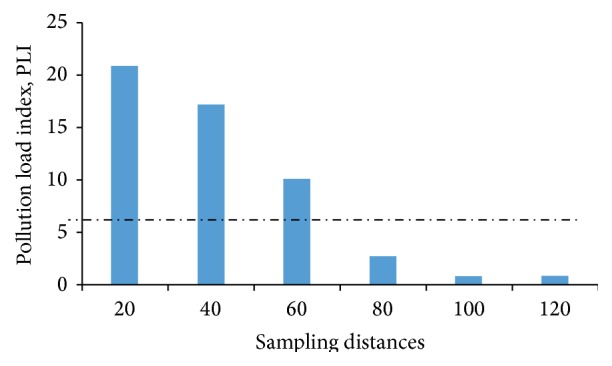
Pollution load index (PLI) values for soil samples.

It is now widely recognized that the measurement of total metal concentration in sediments/soils is not sufficient to provide information about the exact dimension of pollution by heavy metals [24]. As such various indices have been formulated to determine levels of contamination or pollution of soils. Using the geoaccumulation and pollution indices, our study of soils within 100 m from the incinerator were highly contaminated with Pb, Zn, and Cr (Figure 2) and soils within 60 m radius from the incinerator were polluted (Figure 3). The study found soils within 20 m to 60 m to be extremely polluted. Moderate pollution was observed at 80 m from incinerator site (Table 2).

Polluted soils have the possibility of causing harm to humans, animals, and plants, and this raises environmental health concerns. While this study did not explore plant and animal exposure to heavy metals, we recall observing the presence of edible crops such as palm and plantains in the vicinity of the incinerator. The incineration area is also not fenced to prevent accessibility to unauthorized persons and animals. There is therefore a possibility of heavy metal exposure to workers, unauthorized persons, and animals that stray to the waste incineration area.

## 4. Conclusion

All heavy metals assessed by this study in incinerator bottom ash were above the permissible limits by the USEPA criteria. The levels observed in the hospital incinerator ash imply the need for ash to be treated before safe disposal. Unfortunately, this is not the situation and ash is dumped in an open pit close to the incinerator. Our study also revealed soils within 60 m radius from the incinerator to be polluted with heavy metals. These metals can leach into ground water or be carried into water bodies through runoffs, be inhaled in dust from the dump area, and bioaccumulate in plants and animals that stray to the dump site. We anticipate that continuous exposure to heavy metals in ash and soil may pose direct health risk to waste workers at the incinerator site and unauthorized persons who come to the waste incineration area and remotely through the consumption of exposed plants and animals that may have accumulated heavy metals in their tissues and water sources contaminated with heavy metals or by the inhalation of heavy metal laden dust from polluted soils or ash. There is therefore the need to improve the waste management practice at the incinerator site to avert further environmental pollution and human exposure to these elements. It is also recommended that health care waste managers be trained in safe handling of incinerator bottom ash and disposal strategies, and regulatory bodies should also monitor and enforce guidelines for bottom ash management in local health facilities.

## Figures and Tables

**Table 1:** Categorization of soil contamination.

*I*_geo_ values	*I*_geo_ class	Designation of soil quality
>5	6	Extremely contaminated
4-5	5	Strongly to extremely contaminated
3-4	4	Strongly contaminated
2-3	3	Moderately to strongly contaminated
1-2	2	Moderately contaminated
0-1		Uncontaminated to moderately contaminated
0	0	Uncontaminated

Source: Müller, 1969 [11].

**Table 2:** Categorization of pollution load index.

PLI values	Soil quality
8–10	Extremely polluted
6–8	Strongly polluted
4–2	Significantly polluted
2–4	Moderately polluted
0–2	Unpolluted to slightly polluted

**Table 3:** Heavy metals detected in bottom ash.

Heavy metals	*N* = items (30 samples)	Mean conc. of metal (mg/kg)	Std. deviation	USEPA allowable limits for waste disposal to landfill
Hg mg/kg	30	0.88	±0.05	0.2
Pb mg/kg	30	143.80	±39.5	5.0
Zn mg/kg	30	16417.69	±195.18	^*∗*^NA
Ag mg/kg	30	28.38	±0.97	5.0
Cr mg/kg	30	99.30	±3.0	5.0
Cd mg/kg	30	7.54	±2.67	1.0

^*∗*^NA: not available.

**Table 4:** Mean concentrations of heavy metals in soils at different sampling points.

Soil sites	Mean concentrations of metals (mg/kg)					
	Hg	Pb	Zn	Ag	Cr	Cd
20	8.41 ± 0.01	334.83 ± 0.06	910.33 ± 0.48	20.30 ± 0.00	42.24 ± 0.01	4.50 ± 0.02
40	7.51 ± 0.02	208.33 ± 0.58	848.97 ± 0.06	16.47 ± 0.06	40.30 ± 0.10	3.53 ± 0.06
60	4.27 ± 0.03	101.33 ± 0.59	502.33 ± 0.58	5.53 ± 0.06	53.67 ± 0.29	2.03 ± 0.06
80	1.74 ± 0.01	32.83 ± 0.06	245.33 ± 0.59	<0.5 ± 0.00	42.33 ± 0.58	1.24 ± 0.01
100	<0.5 ± 0.0	15.53 ± 0.04	17.00 ± 0.00	<0.5 ± 0.00	14.63 ± 0.23	0.25 ± 0.01
1200	0.50 ± 0.02	4.85 ± 0.10	15.00 ± 0.50	<0.5 ± 0.00	2.32 ± 0.12	0.5 ± 0.01
^*∗*^Allowable limits	0.3	100	70	NA	75.0	3.0

^*∗*^Allowable levels of heavy metals in soils.

NA: not available.

**Table 5:** Correlation matrix of heavy metals in soil.

Variables	Hg	Pb	Zn	Ag	Cr	Cd
Hg	1					
Pb	0.960	1				
Zn	0.979	0.944	1			
Ag	0.992	0.976	0.956	1		
Cr	−0.573	0.538	0.711	0.546	1	
Cd	0.984	0.981	0.984	0.988	0.637	1

Correlation coefficient significant at 95% confidence interval.
